# Sildenafil Reduces Neointimal Hyperplasia after Angioplasty and Inhibits Platelet Aggregation via Activation of cGMP-dependent Protein Kinase

**DOI:** 10.1038/s41598-019-44190-7

**Published:** 2019-05-23

**Authors:** Han-Mo Yang, Sooryeonhwa Jin, Hyunduk Jang, Ju-Young Kim, Joo-Eun Lee, Joonoh Kim, Hyo-Soo Kim

**Affiliations:** 1 National Leading Laboratory for Stem Cell Research, Seoul National University College of Medicine, Seoul, Korea; 2 Department of Internal Medicine, Seoul National University Hospital, Seoul, Korea; 3 Department of Molecular Medicine and Biopharmaceutical Sciences, Graduate School of Convergence Science and Technology, Seoul National University, Seoul, Korea

**Keywords:** Restenosis, Mechanisms of disease

## Abstract

Sildenafil is known to reduce cardiac hypertrophy through cGMP-dependent protein kinase (cGK) activation. Studies have demonstrated that cGK has a central switching role in modulating vascular smooth muscle cell (VSMC) phenotype in response to vascular injury. Here, we aimed to examine the effects of cGK activation by sildenafil on neointimal formation and platelet aggregation. After vascular injury, neointimal hyperplasia in rat carotid arteries was significantly reduced in the sildenafil-treated group. This effect of sildenafil was accompanied by the reduction of viability and migration of VSMCs. Further experiments showed that the increased cGK activity by sildenafil inhibited platelet-derived growth factor-induced phenotype change of VSMCs from a contractile form to a synthetic one. Conversely, the use of cGK inhibitor or gene transfer of dominant-negative cGK reversed the effects of sildenafil, increasing viability of VSMCs and neointimal formation. Interestingly, sildenafil significantly inhibited platelet aggregation induced by ADP or thrombin. This effect was reversed by cGK inhibitor, suggesting that sildenafil inhibits platelet aggregation via cGK pathway. This study demonstrated that sildenafil inhibited neointimal formation and platelet aggregation via cGK pathway. These results suggest that sildenafil could be a promising candidate for drug-eluting stents for the prevention of both restenosis and stent thrombosis.

## Introduction

Phosphodiesterase 5 (PDE5) inhibitors such as sildenafil and tadalafil have known benefits in pulmonary arterial hypertension and erectile dysfunction^1–3^. Moreover, results from preclinical studies have indicated that PDE5 inhibitors regress cardiac hypertrophy, improve contractile function in heart failure (HF), and reduce myocardial infarct (MI) size^4–7^. Although there are still safety issues concerning PDE5 inhibitor use in patients with coronary artery disease, recent studies have shown that the use of PDE5 inhibitors, even in a post-MI cohort, reduced mortality and HF hospitalizations^8^.

Despite the favorable effects of PDE5 inhibitors on the myocardium itself, there have been no studies showing the effects of PDE5 inhibitors in the vasculature, especially on restenosis after percutaneous coronary intervention (PCI). After vascular injury by stent implantation, components including vascular smooth muscle cells (VSMCs) and platelets could be activated, leading to restenosis and stent thrombosis^9–11^.

In cases of restenosis after stent implantation, the status of VSMCs is the most important factor in modulating neointimal formation^9^. VSMCs can be divided into two types, contractile and synthetic^12,13^. Vessel injury such as stent implantation converts VSMCs from the contractile form to the synthetic one^13^. Several studies have shown that cyclic guanosine monophosphate (cGMP)-dependent kinase (cGK) plays a key role in modulating VSMC phenotype and neointimal formation^14^. Moreover, stent thrombosis remains a problem that requires further strategies in the drug-eluting stent (DES) era. cGK, a main pathway of PDE5 inhibitors, is highly expressed in platelets^15^. Thus, we aimed to evaluate the effect of cGK on restenosis and platelet function.

Among the drugs that modulate cGK activity, we chose sildenafil as a potential candidate drug for DES. Sildenafil, an inhibitor of cGMP-specific PDE5, increases intracellular concentration of cGMP, resulting in an enhanced cGK pathway. In this study, we examined the effects of sildenafil on neointimal formation after balloon injury and its mechanisms of action in VSMCs and platelets.

## Results

### PDE5 and cGK were expressed specifically in the smooth muscle cell layer of the vessel wall

**Figure 1:**
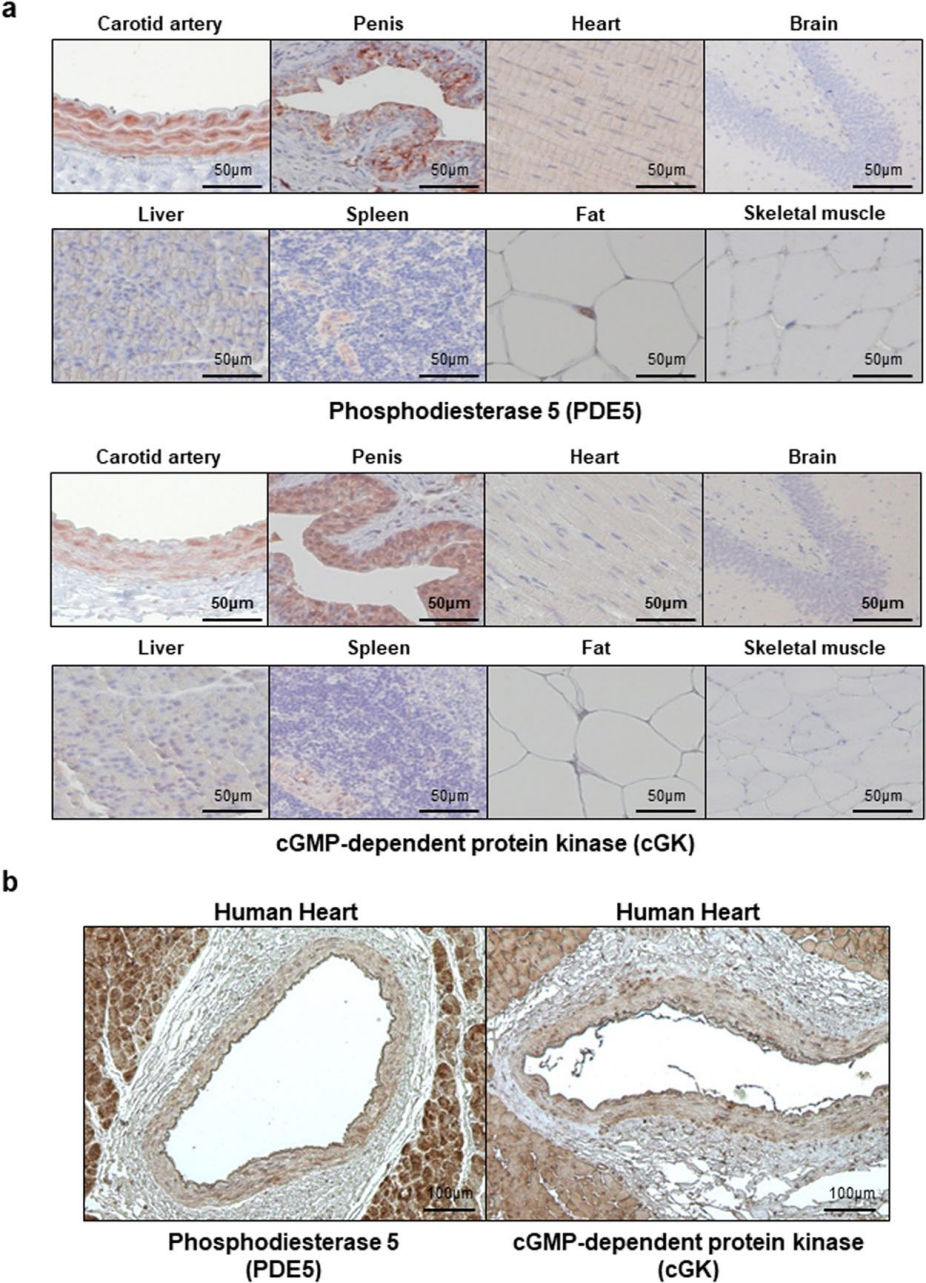
Expression of PDE5 and cGK in the vessel wall. (**a**) Representative figures of the expression patterns of PDE5 and cGK in various organs using immunohistochemistry. Strong expression of both PDE5 and cGK were observed in the smooth muscle cell layer of the vessel wall and in the rat penile tissue. Compared to the intensity of these markers, the heart showed weaker expression (n = 3). (**b**) In the human heart, the smooth muscle layer of the coronary artery strongly expressed both PDE5 and cGK (n = 10).

To see whether sildenafil could affect the vessel wall, we measured the distribution of PDE5 and cGK in various rat organs (Fig. 1a). We found that PDE5 was expressed strongly in the VSMC layer. Penile tissue, used as a positive control, showed an increased level of PDE5, while other tissues such as fat, skeletal muscle, and brain did not. Interestingly, the rat heart tissue showed weak expression of PDE5 as was shown in the previous studies^4^. The distribution of cGK expression in rat tissue was similar to that of the PDE5 distribution. In addition, even in the human heart, increased levels of PDE5 and cGK were observed in the VSMC layer (Fig. 1b). Based on these results, we inferred that sildenafil treatment could regulate the cGK level specifically in the vessel wall.

### Sildenafil reduced neointimal hyperplasia after angioplasty *in vivo*

**Figure 2:**
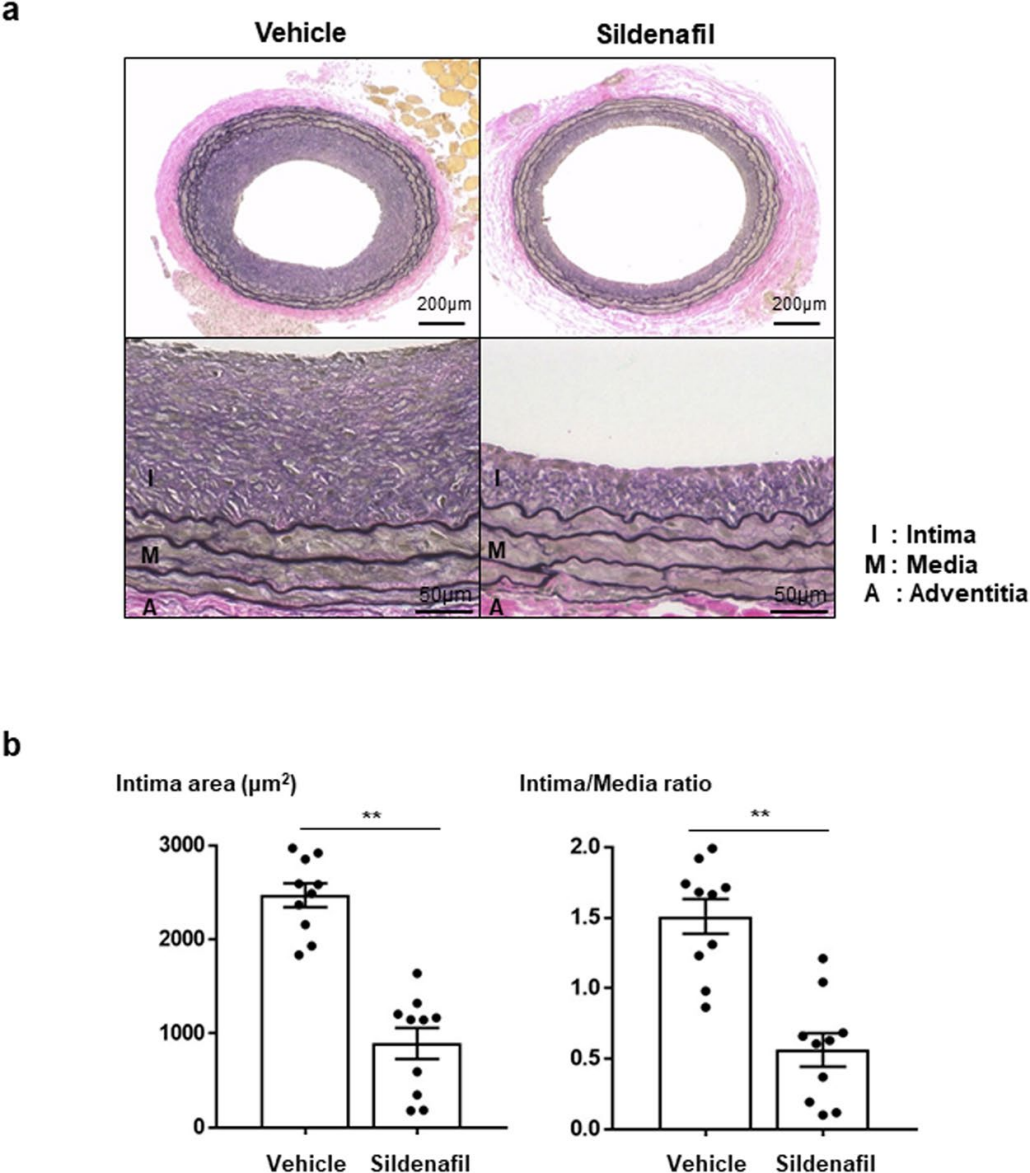
Inhibitory effect of sildenafil on neointimal formation after angioplasty. (**a**) Representative figures of the vessels at 2 weeks after balloon injury in rat carotid arteries (n = 10). Compared to the vehicle-treated group, the sildenafil-treated group significantly reduced neointimal hyperplasia. I = Intima, M = Media, A = Adventitia. Scale bar = 200 µm. (**b**) Quantitative graphs of Intima to Media (I/M) ratio and intimal area at 2 weeks after balloon injury. Graphs show significantly reduced I/M ratio and intimal area in the sildenafil-treated group (n = 10). **P < 0.01.

Next, we examined whether sildenafil could suppress neointimal formation after vascular injury *in vivo* (Fig. S1A). In a rat carotid balloon injury model, administration of sildenafil significantly reduced the development of neointimal formation after 2 weeks, compared with the control group (Fig. 2a). Quantitative analysis showed a significant reduction in Intima area (vehicle-treated group vs. sildenafil-treated group, 2470.2 ± 19.95 vs. 892.6 ± 022.81 μm^2^, respectively, *P* < 0.01) (Fig. 2b).

### Sildenafil reduced proliferation and migration of VSMCs

**Figure 3:**
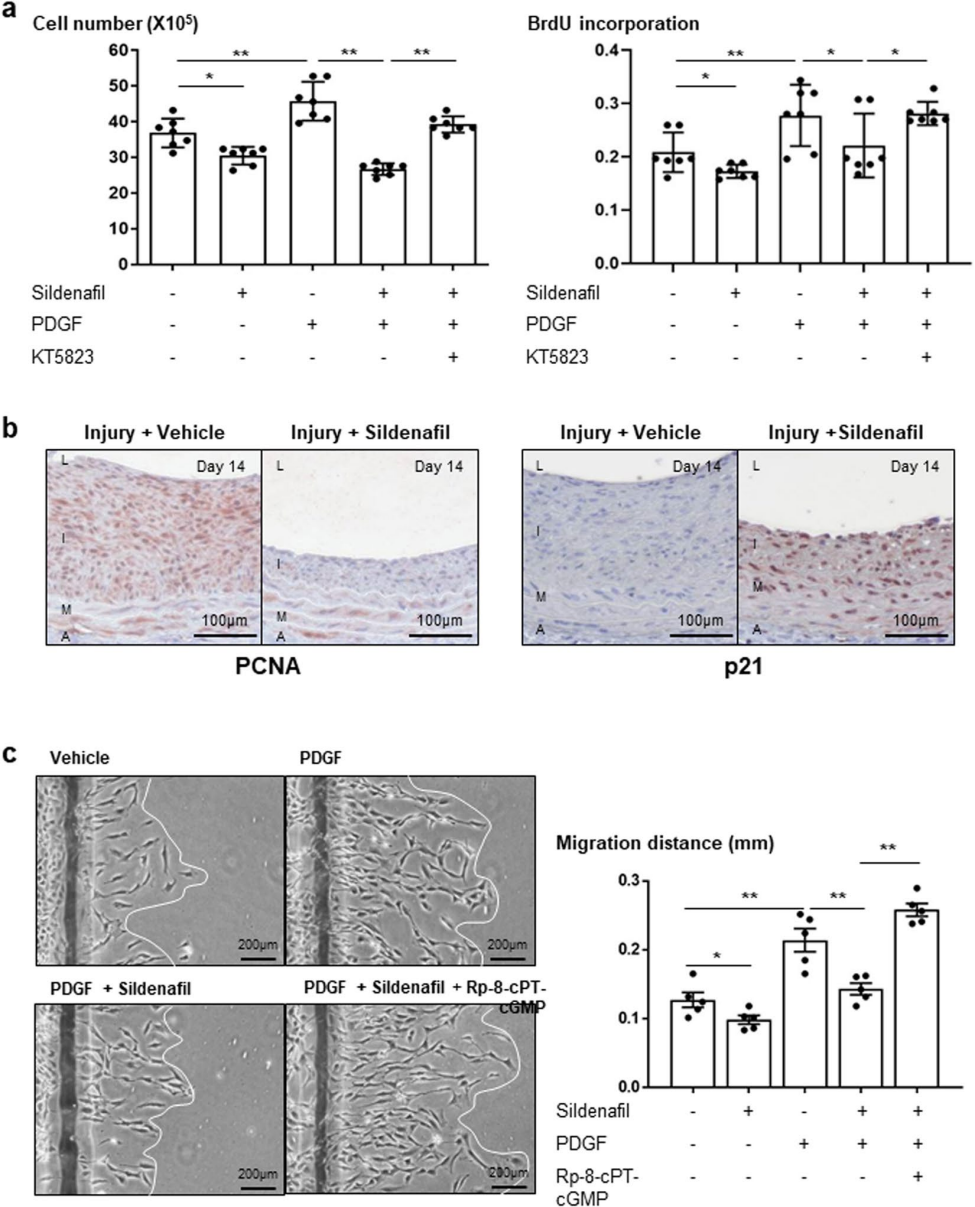
Inhibitory effect of sildenafil on proliferation and migration of VSMCs i*n vitro* and *in vivo*. (**a**) Tryphan blue exclusion assay and BrdU incorporation assay demonstrated that sildenafil treatment reduced the proliferation of VSMCs, which was reversed by the cGK inhibitor (KT5823). cGK inh. = cGK inhibitor. * and ** indicate *P* < 0.05, **P < 0.01 respectively. (n = 7) (**b**) Immunohistochemical staining for PCNA (n = 6) or p21 (n = 3) expressions in the carotid artery wall. Sildenafil treatment decreased PCNA-positive cells and increased p21-positive cells in the vessels. Scale bar = 100 µm. (**c**) Wound migration assay shows that sildenafil treatment significantly inhibited VSMC migration (n = 5). Moreover, this effect was reversed by the cGK inhibitor (Rp-8-cPT-cGMP), suggesting that the inhibitory effect of sildenafil on proliferation and migration of VSMCs might be mediated via the cGK pathway. Scale bar = 100 µm. (**d**) Quantification graph of migration assay. ** indicates *P* < 0.01.

To investigate the cause of the reduced neointimal formation in the sildenafil-treated group *in vivo*, we evaluated the effect of sildenafil on rat VSMC viability. As shown in Fig. 3a, platelet-derived growth factor (PDGF) treatment showed increased viability of VSMCs in a tryphan blue exclusion assay. Sildenafil treatment effectively reduced VSMC viability. The BrdU incorporation assay showed that sildenafil treatment also effectively reduced cell proliferation (Fig. 3a). Interestingly, treatment with the cGK inhibitor (KT5823) reversed sildenafil’s effect on the viability and proliferation of VSMCs, indicating that the effects of sildenafil might be mediated via the cGK pathway. In the injured arteries, sildenafil treatment decreased the proliferating cell nuclear antigen (PCNA)-positive cell number and increased the p21-positive cell number (Fig. 3b). The migration of VSMCs plays an important role in the process of neointimal formation. Therefore, we measured the effect of sildenafil on VSMC migration. Sildenafil treatment reduced the migration of VSMCs, which was reversed by the cGK inhibitor (Rp-8-cPT-cGMP) (Fig. 3c). These data indicated that the inhibitory effect of sildenafil on proliferation and migration of VSMCs could contribute to the reduction of neointimal formation after vascular injury.

### Sildenafil regulated cGK activity and modulated VSMC phenotype

**Figure 4:**
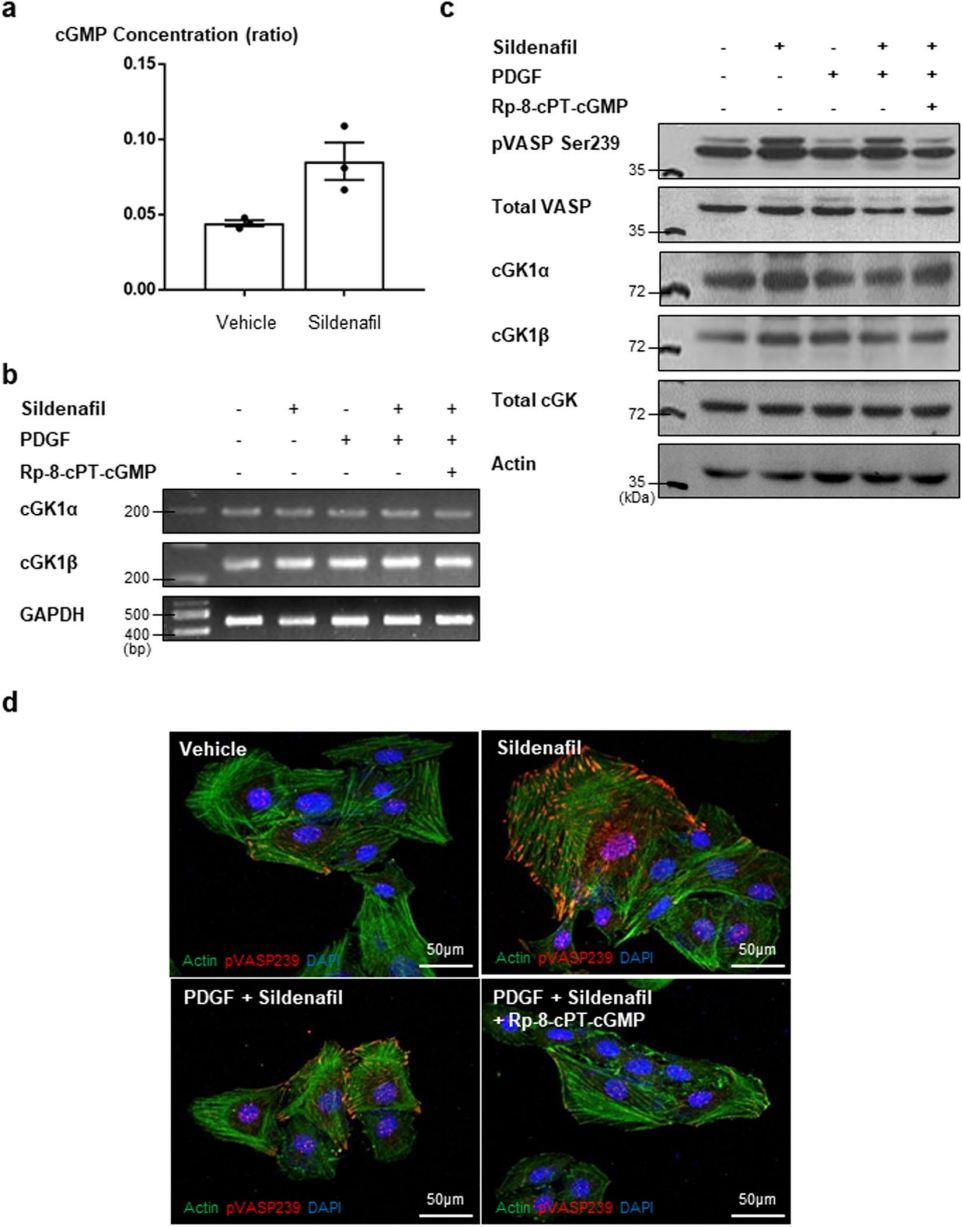
Effects of sildenafil on cGK activity. (**a**) Enzyme immunoassay for cGMP shows that the concentration of cGMP increased in VSMCs with sildenafil treatment. *P* values is 0.05 between two groups (n = 3). (**b**) RT-PCR shows that sildenafil treatment did not change the expression cGK Ια and cGK Ιβ. cGK inh. = cGK inhibitor (n = 3). (**c**) Western blot analysis indicates that sildenafil treatment increased VASP phosphorylation (a substrate of cGK), but did not change the level of cGK itself. The phosphorylation of VASP by sildenafil was reversed by cGK inhibitor. pVASP Ser 239 = phospho-VASP at serine239 (n = 3). (**d**) Immunofluorescence staining for pVASP shows the similar effect with the previous western blot. pVASP239 = phospho-VASP at serine239 (n = 3). Scale bar = 50 µm.

**Figure 5:**
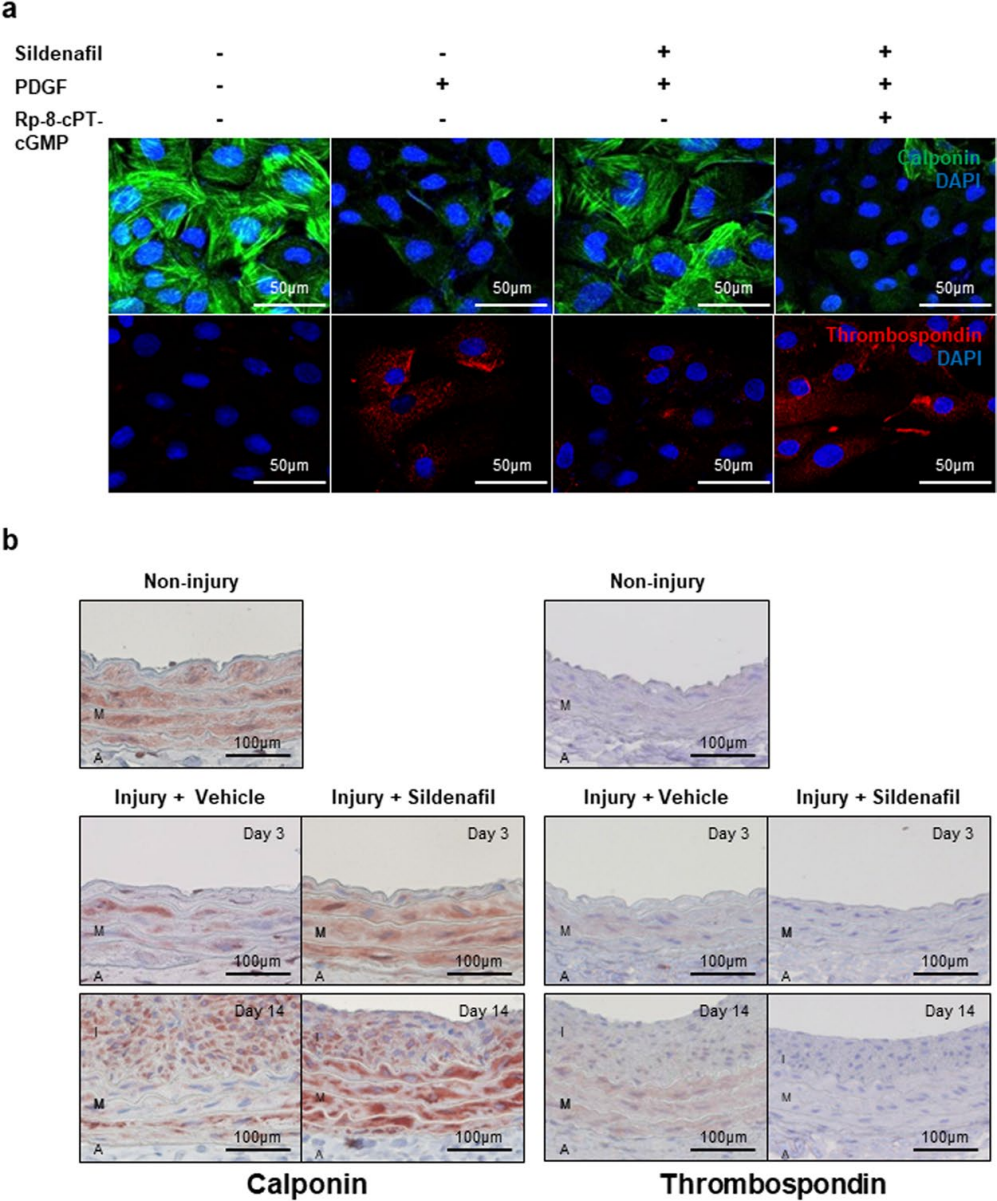
Effects of sildenafil on VSMC phenotype modulation. (**a**) Immunofluorescence staining for calponin and thrombospondin. PDGF treatment increased the level of thrombospondin (a marker of synthetic form of VSMC) and decreased the level of calponin (a marker for contractile form). Sildenafil treatment inhibited the effect of PDGF on these markers. Finally, cGK inhibitor reversed the effect of sildenafil, suggesting that sildenafil could modulate VSMC phenotype via the cGK pathway. Scale bar = 50 µm. cGK inh. = cGK inhibitor (n = 3). (**b**) Immunohistochemical staining for calponin and thrombospondin. The arterial wall at 3 days and 2 weeks after injury showed that sildenafil treatment elevated the expression of calponin and reduced the level of thrombospondin. Scale bar = 100 µm (n = 3).

Because sildenafil is a PDE5 inhibitor which increases the level of cGMP, we examined whether sildenafil could increase the level of cGMP in VSMCs. Furthermore, we measured whether sildenafil treatment could increase cGK activity or cGK expression. Results showed that sildenafil treatment increased the concentration of cGMP by twofold (Fig. 4a). RT-PCR showed that mRNA expression of cGK Ια and cGK Ιβ was not changed by sildenafil treatment (Figs 4b and S2a). However, western blot analysis and immunofluorescence staining of phospho-vasodilator-stimulated phosphoprotein (VASP) (a substrate of cGK) showed that sildenafil treatment increased the activity of cGK (Figs 4c,d and S2b). Conversely, cGK inhibitor (Rp-8-cPT-cGMP) treatment reversed the effect of sildenafil on phospho-VASP. These data indicated that sildenafil regulates the kinetic activity of cGK, and not its expression. It is known that cGK activity is associated with the phenotypic changes of VSMC^14^. Based on this, we hypothesized that sildenafil could modulate the VSMC phenotype. The level of calponin, a marker for the contractile form of VSMC, was examined by immunofluorescence staining. PDGF stimulation reduced calponin expression, which was reversed by sildenafil treatment (Figs 5a and S2c). In the case of thrombospondin, a marker of synthetic form, we observed the opposite change (Fig. 5a). This suggests that sildenafil can modulate the VSMC phenotype. Immunohistochemistry for these markers in the injured vessel wall showed similar results (Fig. 5b).

### The effect of sildenafil on VSMCs and neointimal formation was mediated through the cGK pathway

**Figure 6:**
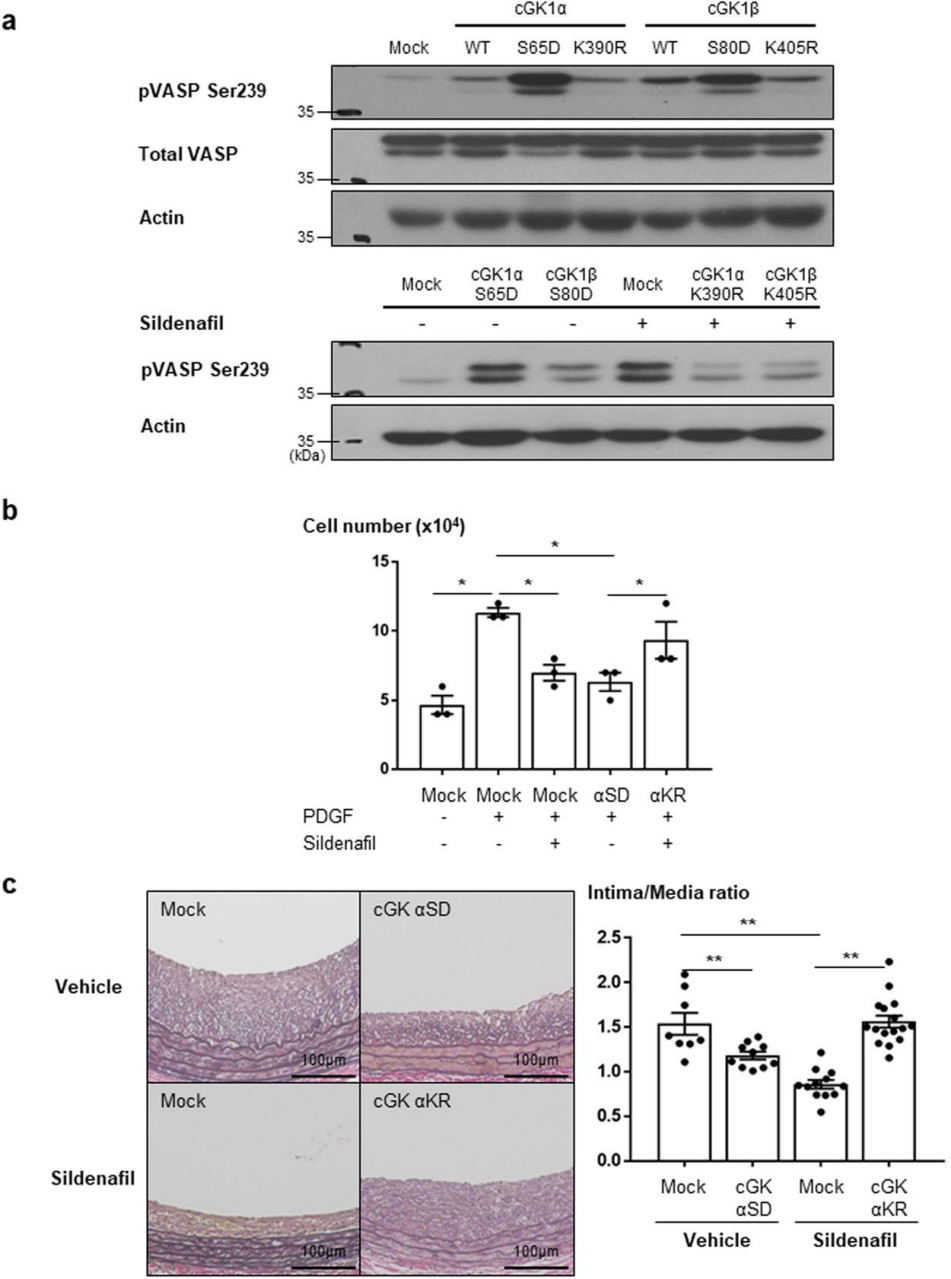
*In vitro* and *in vivo* effects of sildenafil mediated via cGK pathway. (**a**) Western blot assay (upper panel) demonstrates the activity of active form (S65D) and dominant negative (K390R) of cGK1 α and β. Lower panel shows that cGK activation by sildenafil was reversed by gene transfer of dominant-negative of α subtype of cGK1. pVASP Ser 239 = phospho-VASP at serine239 (n = 3). (**b**) The graphs show that the inhibitory effect of sildenafil on cell viability was reversed by the gene transfer of dominant-negative of cGK1α (αKR). The gene transfer of active form of cGK1α (αSD) reduced the increased viability by PDGF treatment (n = 3). * indicates *P* < 0.05. (**c**) *In vivo* sections at 2 weeks after injury demonstrated that the transfer of lentiviral vector expressing active form of cGK(αSD) showed a similar effect as sildenafil-treated group, which was reversed by the transfer of lentiviral vector expressing dominant-negative form of cGK (αKR) (n = 10). Scale bar = 100 µm. ** indicates *P* < 0.01.

To test whether sildenafil acts through the cGK pathway, we conducted several additional experiments with cGK inhibitor or gene transfer of dominant-negative (inactive form) cGK. As already shown in Fig. 3a, cGK inhibitor reversed sildenafil-induced cGK activation in terms of VSMCs viability and proliferation. We performed *in vitro* and *in vivo* studies with gene transfer of dominant-negative (KR form) or active (SD form) of cGK. Gene transfer of active or dominant-negative of cGK effectively modulated cGK activity (Figs 6a and S2d). Moreover, western blot analysis for phospho-VASP demonstrated that sildenafil-induced cGK activation was reversed by gene transfer of the dominant-negative cGK (Fig. 6a lower panel), especially with α subtype of cGK. Gene transfer of active cGK reversed the increased number of VSMCs by PDGF treatment. In addition, the cell number reduced by sildenafil was reversed by the gene transfer of dominant-negative cGK (Fig. 6b). This indicates that sildenafil regulates the viability of VSMCs through the cGK pathway. An *in vivo* experiment demonstrated that gene transfer of the active form of cGK showed a similar result as sildenafil treatment, and that gene transfer of dominant-negative cGK reversed the effect of sildenafil, suggesting that sildenafil reduced neointimal formation via the cGK pathway (Fig. 6c).

### Sildenafil treatment inhibited platelet aggregation via the cGK pathway

**Figure 7:**
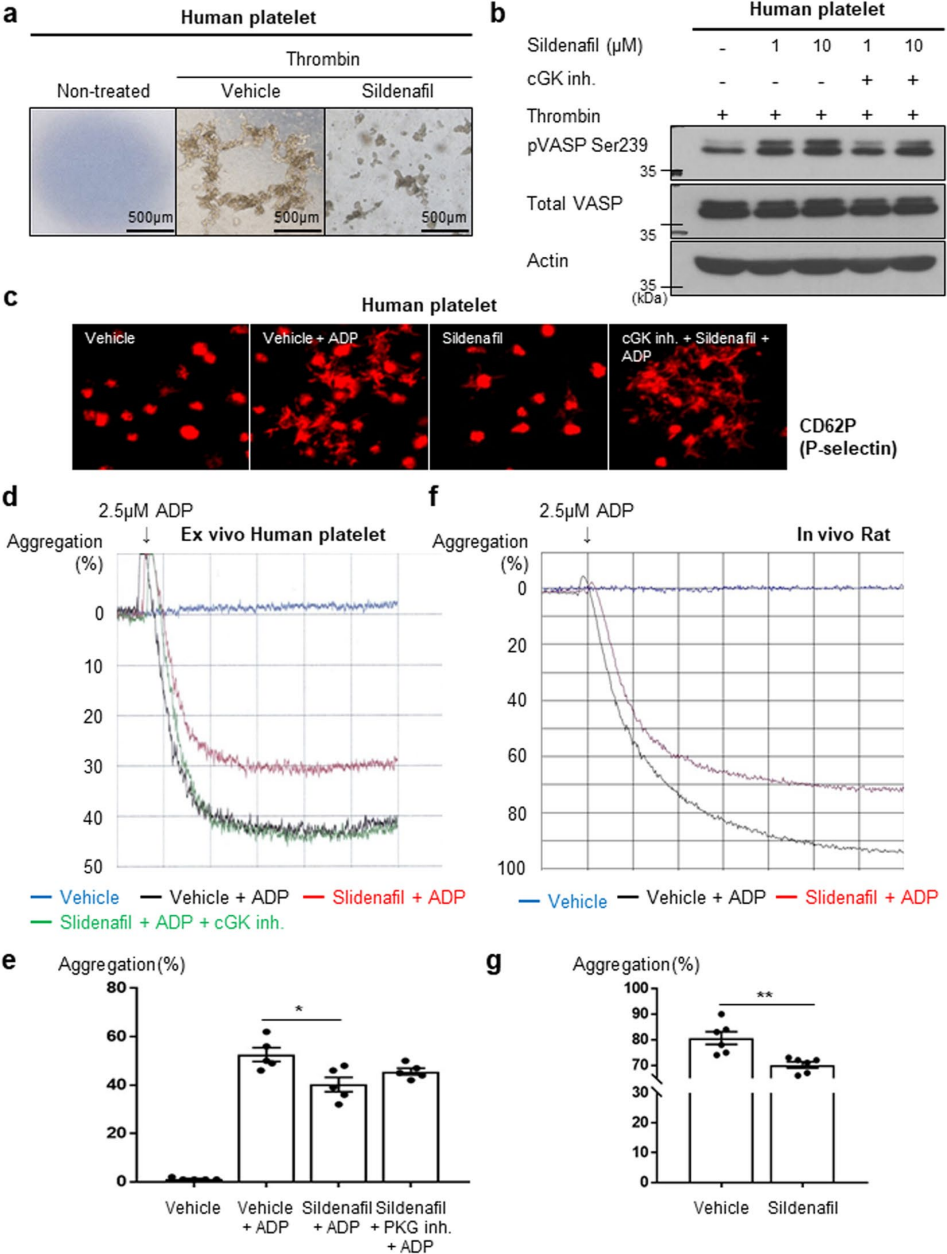
Inhibition of platelet aggregation by sildenafil. (**a**) Sildenafil treatment inhibited the aggregatory effect of thrombin on human platelets. Scale bar = 500 µm. (**b**) Western blot for VASP phosphorylation (indicates inhibitory activity of platelet aggregation) shows that the increased VASP phosphorylation by sildenafil was reversed by cGK inhibitor in human platelets (n = 3). cGK inh. = cGK inhibitor. pVASP Ser 239 = phospho-VASP at serine239. (**c**) Immunofluorescence staining for P-selectin(C62P), an activated platelet marker, shows that the aggregation of platelets induced by ADP was inhibited by sildenafil treatment. Moreover, this effect of sildenafil was reversed by cGK inhibitor, suggesting that sildenafil could inhibit platelet aggregation through the cGK pathway (n = 3). (**d**) Platelet aggregometer with platelet rich-plasma from a human donor showed that cGK activation by sildenafil treatment reduced ADP-induced platelet aggregation. In addition, this reduced platelet aggregation by sildenafil was reversed by cGK inhibitor (n = 5). (**e**) Quantitative graph of *ex vivo* aggregation study suing human platelets (n = 5). * indicates *P* < 0.05. (**f**) Representative figure of platelet aggregometer with platelet rich-plasma from rat peripheral blood treated with vehicle or sildenafil for 2 weeks (n = 6). (**g**) The quantitative graph also shows that sildenafil treatment inhibited platelet aggregation *in vivo* (n = 6). ** indicates *P* < 0.01.

To test whether the cGK pathway is associated with platelet aggregation, platelets were treated with sildenafil or sildenafil plus cGK inhibitor (Rp-8-cPT-cGMP). Sildenafil treatment reduced the aggregation of human platelets induced by thrombin (Fig. 7a). As shown in Figs 7b and S2e, sildenafil increased the phosphorylation of VASP (a marker for inhibition of platelet aggregation), which was reversed by cGK inhibitor. Also, the immunofluorescence staining for P-selectin, an activated platelet marker, showed that sildenafil could modulate platelet aggregation via the cGK pathway (Fig. 7c). In *ex vivo* experiments with human platelets, this effect was demonstrated in a quantitative manner (Fig. 7d,e). The *in vivo* rat study demonstrated that ADP-induced platelet aggregation in the sildenafil-treated group was reduced compared to that of the vehicle-treated group (Fig. 7f,g). These results suggest that sildenafil can inhibit platelet aggregation via the cGK pathway.

## Discussion

**Figure 8:**
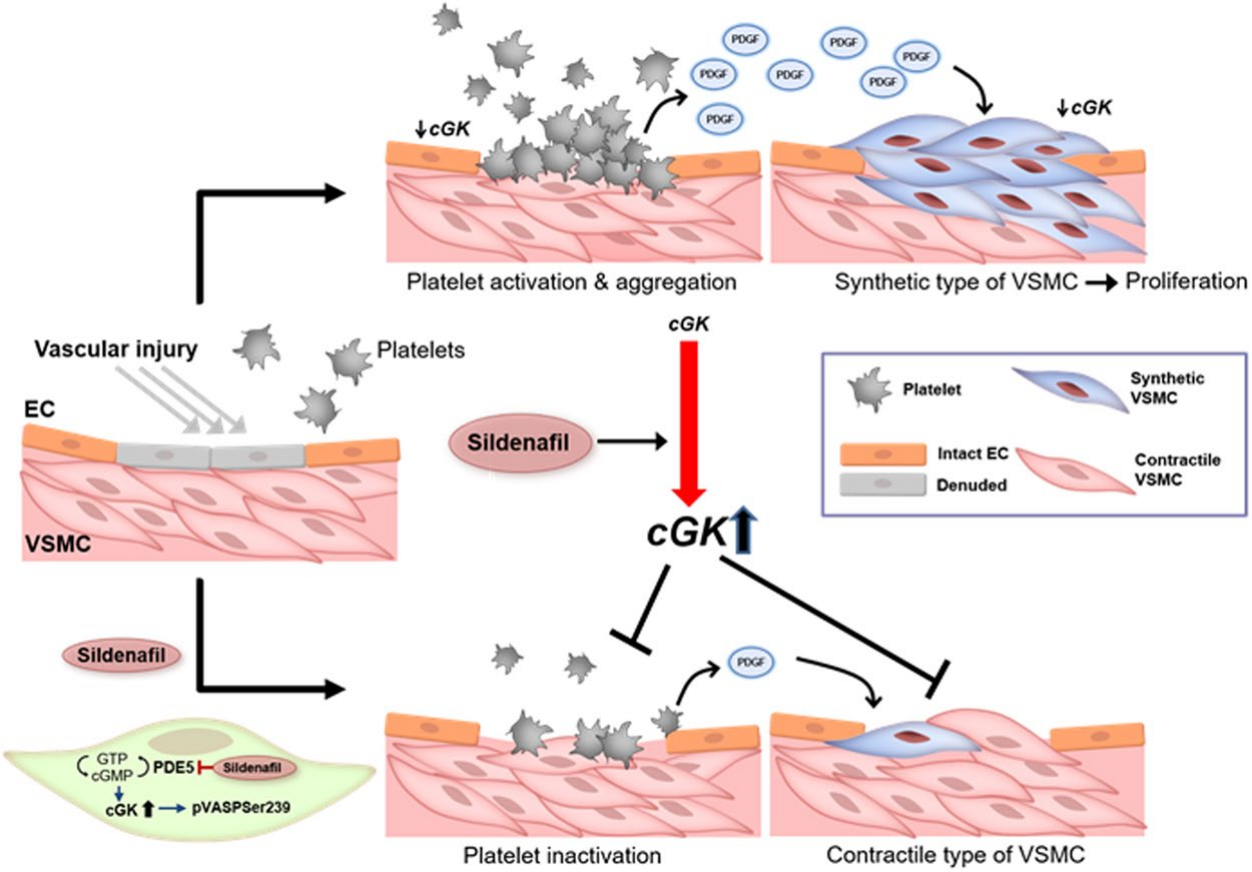
Schematic figure of the role of cGK and sildenafil treatment in the vessel and platelets. After vascular injury such as balloon angioplasty or stent implantation, endothelial cells (ECs) are denuded, resulting in the downregulation of nitric oxide. Subsequently, the level of cGK in VSMCs and platelets decreases. VSMCs are converted to a synthetic form from a contractile form by the decreased cGK activity. Furthermore, the decreased cGK in platelets facilitates platelet aggregation. In addition, activated platelets produce PDGF which can stimulate VSMC proliferation. In contrast, sildenafil treatment can prevent the decrease of cGK activity in VSMCs and platelets, leading to the inhibition of both neointimal growth and platelet aggregation.

In vascular biology in the DES era, VSMCs and platelets have become very important factors in terms of regulating restenosis and stent thrombosis. In the present study, *in vitro* experiments showed that activation of cGK by sildenafil modulated VSMC phenotype, resulting in the reduction of VSMC proliferation and migration. In addition, *in vivo* experiments demonstrated that sildenafil regulated VSMC phenotype and reduced neointimal formation after injury via cGK activation. Finally, cGK activation by sildenafil also inhibited platelet aggregation. Therefore, sildenafil could be a candidate drug satisfying the requirements for ideal DES (Fig. 8).

### Distribution of PDE5 and cGK and their effect on cardiovascular diseases

Of all the PDEs catalyzing the breakdown of the cAMP and cGMP, PDE5 is the main cGMP-handling enzyme^16,17^. Several PDEs such as PDE 2,5,6, and 9 are known to be specific for cGMP. However, PDE 2, 6 and 9 are distributed mainly in adrenal gland, photoreceptor or pigment epithelial cells^18^. Moreover, other PDEs such as PDE3 are known to regulate the level of both cAMP and cGMP. However, they have a substrate preference for cAMP over cGMP. A PDE3 inhibitor, cilostazol, has shown its effects mainly by the upregulation of cAMP and protein kinase A. PDE5 is expressed in vascular/bronchial smooth muscle cells as well as in platelets^15,19^. Our study showed that cGK had the same distribution as PDE5. Therefore, sildenafil treatment could be specifically effective in reducing restenosis and inhibiting platelet aggregation with a low risk of adverse events in other organs. Owing to the distribution of PDE5 in the pulmonary artery, sildenafil appears to be effective in treating pulmonary arterial hypertension, leading to the reduction of preload of the right ventricle. As shown in Fig. 1, even in the human myocardium, the expression of PDE5 and cGK was observed. Therefore, sildenafil could have direct myocardial effects in diverse processes of cardiac remodeling including cardiac hypertrophy and post-MI apoptosis.

### Sildenafil inhibits platelet aggregation

Over the past decades, great progression has been made with coronary stents and antithrombotic therapies. However, stent thrombosis remains a problematic issue in the DES era. Sometimes, intensive anti-platelet therapy should be given based on the individual characteristics of patients or coronary lesions. In those cases, intensive antithrombotic therapy may cause fatal bleeding. Considering this fact, coating stents with a drug which has an antithrombotic effect would reduce not only risk of stent thrombosis, but also the side effects such as bleeding caused by systemic use of antiplatelet drugs. In our study, sildenafil reduced the aggregation of platelets induced by thrombin and ADP. From the results of our experiments with P-selectin and VASP phosphorylation which is known to be the best marker for inhibition of platelet aggregation^20,21^, we concluded that sildenafil modulated platelet aggregation via the cGK pathway. Furthermore, the *in vivo* rat study and the *ex vivo* human platelet study showed that sildenafil could effectively reduce platelet aggregation. There have been some conflicting results regarding the effect of sildenafil on platelet aggregation^22–24^. One study showed that cGMP-dependent protein kinase might show biphasic effect, initially transient stimulation and subsequently inhibition^24^. It might be dependent on the patient’s status or presence of other stimulatory factors. Under our experimental condition such as *ex vivo* or *in vivo* experiments, sildenafil could effectively inhibit platelet aggregation. Considering the results of our *in vivo* study, sildenafil should be effective in inhibiting platelet aggregation after multiple doses of sildenafil.

### Effect of sildenafil on diverse underlying mechanisms of restenosis in the current DES era

Besides the inhibitory ability of VSMCs proliferation, the ability to inhibit VSMCs migration is also important in reducing neointimal hyperplasia. The phosphorylation of VASP at Ser239 is associated with the anti-migratory effect. Because sildenafil treatment increased VASP phosphorylation at Ser239, we can assume that it might have the potent inhibitory effect on the migration of VSMCs. In our study, migration assay demonstrated that sildenafil treatment inhibited the migration capacity of VSMCs, resulting in reducing neointimal hyperplasia.

In the process of neointimal formation after angioplasty, both VSMCs and endothelial cells (ECs) play an important role. Rapid re-endothelialization is needed to reduce both neointimal hyperplasia and thrombus formation. However, the drugs used for contemporary DES repress the viability of both VSMCs and ECs. Until now, many strategies have been suggested to overcome this hurdle. Among them, one study reported the interesting result using a selective microRNA-based approach, resulting in inhibiting VSMC proliferation while preserving ECs^25,26^. Our previous paper also reported that activation of cGK pathway using exisulind showed its effect in reducing both restenosis and platelet aggregation^27^. In this study, the treatment of exisulind could increase re-endothelialization by facilitating the differentiation of endothelial progenitor cells into ECs. However, in terms of the mechanism of exisulind, this drug could inhibit neointimal formation not only by activation cGK pathway, but also by activation JNK pathway, resulting in the increase of VSMC apoptosis. In our study, we did not check whether sildenafil treatment affect the viability of ECs, which could affect thrombus formation after injury. Although sildenafil treatment inhibited thrombus formation in our study, further study is required to verify this issue.

### Revisit of sildenafil in cardiovascular disease

Cilostazol is a PDE3 inhibitor known to induce vasodilation and to reduce platelet aggregation^28^. In spite of showing dramatic efficacy in preclinical studies and small-sized clinical studies, cilostazol failed to show beneficial effects in reducing major adverse cardiac events in recent randomized trials^28–30^. Although there are concerns about the safety of sildenafil use in patients with coronary artery disease due to the risk of coronary steal or hypotension, several studies have shown the benefits of sildenafil in many aspects. Sildenafil has been used extensively for the treatment of pulmonary arterial hypertension, as well as erectile dysfunction^1–3^. Several preclinical and clinical studies have shown evidences that sildenafil regresses left ventricular hypertrophy, reduces infarct size in myocardial infarction, improves heart function in heart failure, and reduces ventricular arrhythmia^4–7,31^. Moreover, recent studies showed that, in 43,145 patients with a first MI, treatment with PDE5 inhibitors reduced the risk of death and HF hospitalizations^8^.

### Clinical applications of our study

The use of new-generation of DES have shown much less rate of restenosis, giving opportunities for cure even in case of high-risk lesions such as ostial lesions^32^. Since there are still difference for each new generation of DES in terms of clinical outcomes^33^, there might be further chances to develop a new candidate drug for DES. Many drugs such as paclitaxel, sirolimus, and zotarolimus could be toxic to human body if adequate concentration of each oral drug for anti-proliferative effect should be reached. However, in terms of ability to show efficacy, the oral dose 50–100 mg of sildenafil used in clinical setting is comparable with the concentration used in our *in vitro* and *in vivo* studies. Therefore, among the drugs given orally, sildenafil could have priority in achieving goals with less toxicity.

The use of first-generation DES showed the increased rate of stent thrombosis, as compared to bare-metal stents^34,35^. However, recent studies demonstrated that contemporary DES use showed no increase or even lower rate of stent thrombosis than bare-metal stents^36^. This might be caused by newer platform of stents or new antiplatelet agents. However, there are still high-risk subgroups for stent thrombosis and restenosis such as patients with complex coronary lesions or with diabetes. Therefore, there have been a lot of efforts to pursue the ideal drugs with the ability to reduce restenosis and stent thrombosis. Considering the effects of sildenafil in reducing restenosis and platelet aggregation, sildenafil could be a promising candidate.

Based on the results of our study, clinical trials could be conducted using stents coated with sildenafil or oral administration of sildenafil after stent implantation. Between two strategies, its effect could be much more potent with fewer side effects when used as a coating drug for DES. As mentioned above, in patients with coronary artery disease, for whom hypotension or other side effects is a concern, the use of a lower oral dose of sildenafil could be suggested. Our study is limited by the use of some animal data. However, in human tissue, especially such as human heart, human coronary artery, and human platelets, we could detect the target molecule of sildenafil and the experiments with human platelets showed promising results. Therefore, if these results could be verified in the clinical trials, sildenafil could be an ideal drug for reducing restenosis and stent thrombosis.

In conclusion, our study showed that sildenafil could be a potent drug in reducing neointimal formation after angioplasty by activating cGK. Moreover, sildenafil inhibited platelet aggregation via the cGK pathway. These findings suggest that sildenafil could be a candidate drug for DES for the prevention of restenosis and stent thrombosis.

## Materials and Methods

The authors declare that all supporting data are available in this article and the Supplementary Material. The authors also declare that we will make study materials and analytic methods available to other researchers on the request from the corresponding author (H. Kim). Detailed experimental methods are described in the Supplementary Material.

All experiments dealing with humans or human products were conducted with informed consent from patients and approved by the Institutional Review Board (IRB) of Seoul National University Hospital. All human research was performed in accordance with the relevant guidelines and regulations. All animal studies were performed after receiving approval from the Institutional Animal Care and Use Committee (IACUC) of Clinical Research Institute in Seoul National University Hospital and complied with the National Research Council (NRC) ‘Guidelines for the Care and Use of Laboratory Animals.’

### Statistical analysis

All data are presented as means ± standard error of means (SEM). The significance of differences between two groups were determined using nonparametric anaysis such as Mann-Whitney test or Wilcoxon signed rank test. SPSS version 21.0 (SPSS Inc., Chicago, IL) was used for the analysis and *P* values of < *0.05* were considered to be statistically significant.

## Supplementary information

Supplementary Material Online

## Data Availability

All data are available in the manuscript or the Supplementary Materials.
