# Supplementary material for: Sildenafil Reduces Neointimal Hyperplasia after Angioplasty and Inhibits Platelet Aggregation via Activation of cGMP-dependent Protein Kinase
Source: Sci Rep. 2019 May 23;9:7769. doi: 10.1038/s41598-019-44190-7 (PMC6533301; doi:10.1038/s41598-019-44190-7)

**Supplementary Material Online**

**Sildenafil Reduces Neointimal Hyperplasia after Angioplasty and Inhibits Platelet Aggregation via Activation of cGMP-dependent Protein Kinase**

* Han-Mo Yang, MD^1,2,3^, * Sooryeonhwa Jin, MS^1,3^, * Hyunduk Jang, PhD^1,3^

Ju-Young Kim, PhD^1,3^, Joo-Eun Lee, PhD^1,3^, Joonoh Kim, MS^1,3^, Hyo-Soo Kim, MD^1,2,3^

^1^National Leading Laboratory for Stem Cell Research, Seoul National University College of Medicine, Seoul, Korea

^2^Department of Internal Medicine, Seoul National University Hospital, Seoul, Korea

^3^Srategic Center of Cell & Bio Therapy, Seoul National University Hospital, Seoul, Korea

* These authors contributed equally to this work.

**Supplementary Materials and Methods**

**Supplementary Figures and Legends**

**Supplementary Materials and Methods**

All experiments dealing with humans or human products were conducted with informed consent and approved by the Institutional Review Board (IRB) of Seoul National University Hospital. All animal experiments were performed after receiving approval from the Institutional Animal Care and Use Committee (IACUC) of Clinical Research Institute in Seoul National University Hospital and complied with the National Research Council (NRC) ‘Guidelines for the Care and Use of Laboratory Animals.’

**Primary cell culture of rat VSMCs**

Primary culture of Vascular Smooth Muscle Cell from rat abdominal artery was performed by 0.2 % collagenase type 2 (Invitrogen) and 0.5 mg/㎖ elastase. Adipose tissue and adventitia that surrounds artery were extracted, and vessels were transfer to fresh DMEM media. Washing in phosphate buffer saline at several times and snipped off the artery. It was incubated in 100 rpm shaking at 37 ℃ for 30 minutes. After centrifuge with a soup, cells were suspended in DMEM/F12 and seeded at 100 mm cell culture dish. To confirm adequate separation, vascular smooth muscle cells were strained with mouse anti-smooth muscle actin (Santa Cruz Biotechnology, 1:2500 dilution).

**Cell viability and proliferation assay.**

Cell viability and proliferation were measured by trypan blue exclusion assay and incorporation of 5-bromo-2-deoxyuridine (BrdU, R&D, 5015) according to manufacturer's instruction.

VSMC were seeded in 6 well multi-plate at 3×10^5^ cells/well in DMEM/F12 (Gibco) and incubated at 37 ℃ in an atmosphere 95 % O_2_ and 5 % CO_2_. Cells were cultured 48 hours at serum starvation media and 10 μM KT5823 (cGK inhibitor, Sigma Aldrich, K1388-100U), 1 μM sildenafil, DMSO (Vehicle) were added for 30 minutes. Then 10 ng/㎖ recombinant rat PDGF (rrPDGF, R&D, 520-BB-050) was treated. 3 days after treatment of rrPDGF, cells were counted using hemocytometer.

At BrdU incorporation assay, cells were seeded in 96 well multi-plate at 3×10^3^ cells/well in DEME/F12 with 10 % FBS. After 2 days, cells were incubated with serum starvation in 48 hours. Samples were treated under the same condition with trypan blue exclusion assay. And 3 hours after treatment of 10 ng/㎖rrPDGF, BrdU was added 1 ㎕ per well. And then measure incorporation ratio of BrdU after 18 hours.

To confirm apoptotic cell death, FACS analysis for Propidium Iodide (PI) was behaved with BD FACS Canto™Ⅱ Flow Cytometer. Cells were harvested 3 days after rrPDGF stimulation.

**Cyclic GMP ELISA assays.**

Cyclic GMP assay was measured using Cyclic GMP Assay kit (R&D system, KGE003) according to previously reported manufacturer's instruction. Cells were seeded 3×10^5^ cells/well, after rrPDGF treatment, soup were harvested at order by time.

**Western Blot Analysis.**

Phosphorylated VASP at Serine 239, total VASP, cGK Ια, cGK Ιβ, total cGK, actin proteins from rat VSMC were extracted in a lysis buffer containing 50 mM Tris (pH 7.2), 250 mM NaCl, 1 % NP40, 0.05 % Sodium Dodecyl Sulfate (SDS), 2 mM Ethylenediaminetetraacetic acid (EDTA), 0.5 % Deoxycholic acid, 10 mM β-glycerol phosphate, 100 mM Sodium fluoride (NaF), 1 mM Orthovanadate, 1 tablet/10 ㎖ of Protease inhibitor cocktail (ROCHE). Samples were loaded to 10 % SDS-PAGE gel, transferred to a PVDF membrane. PVDF membrane was probed the indicated primary antibody and appropriate secondary antibodies conjugated with horseradish peroxidase (HRP). Then, membrane was detected by ECL reagent (GE Healthcare). Rabbit anti-pVASP Ser239 antibody (1:2500 dilution, Cell signaling, #3114S), rabbit anti-total VASP antibody (1:2500 dilution, Cell signaling, #3112S), goat anti-cGKIα antibody (1:2500 dilution, Santa Cruz Biotechnology, sc-10335), goat anti-cGKΙβ antibody (1:2500 dilution, Santa Cruz Biotechnology, sc-10341), goat anti-β Actin antibody (1:2500 dilution, Santa Cruz Biotechnology, sc-1616). Secondary antibodies were donkey anti-rabbit IgG-HRP (1:2500 dilution, Santa Cruz Biotechnology, sc-2313), donkey anti-goat IgG-HRP (1:2500 dilution, Santa Cruz Biotechnology, sc-2020). Because some antibodies were from same source, proteins were detected at diverse membranes. Membrane images were analyzed by Image Pro software.

**Reverse Transcription Polymerase Chain Reaction.**

Messenger RNA was harvest by Trizol method. Complementary DNA (cDNA) synthesis was performed using Invitrogen Power cDNA synthesis kit. Specific primer for cGKIα and

cGKIβ were used. Primer for cGKIα forward: GCTCAAGGAGGAGAGGATCA, cGKIα reverse: AGGTCGTGGAAGGACCTGTA, cGKIβ forward: GCTCAAGGAGGAGAGGAT CA, cGKIβ reverse: AGGTCGTGGAAGGACCTGTA. Denaturation temperatures of all primers are 58 Celsius degrees.

**Immunofluorescence staining.**

For immunofluorescence staining, rat VSMCs are seeded 2×10^5^ cells/well in coverglass-bottom dish (SPL Lifescience). Fixed with 100 % methanol at -20 ℃, 10 minutes and probed with indicated primary antibody; Mouse anti-calponin (1:200 dilutions, Sigma Aldrich, C2687), rabbit anti-thrombospondin antibody (1:250 dilution, Santacruz, sc-14013), rabbit anti-pVASP Ser239 (1:200 dilution, Cell Signaling, #3114S), nucleic acid staining with 4', 6’-diamidino-2-phenylindole (DAPI, 1:5000 dilutions). Secondary antibodies were Alexa flour donkey anti-mouse 488 (1:1000 dilution, Invitrogen, A21202), Alexa flour donkey anti-rabbit 555 (1:1000 dilution, Invitrogen, A31572). The cells were mounted with fluorescent mounting medium (DAKO, S3023) and viewed with a confocal microscope Carl Zeiss LSM710 (Carl Zeiss, Germany)

**In vitro Migration assay.**

After incubated in 10 % FBS with DEME/F12 media, and had a serum starvation. 1 % Thymidine was added to inhibit cell proliferation. After serum starvation, 10 μM Rp-8-cPT-cGMP (cGK inhibitor, Cayman, 221905-35-7), 1μM sildenafil were added for 30 minutes, and stimulated with 10 ng/㎖rrPDGF. Cells were observed at 24 hours, 48 hours and took a picture of moved cells against base line. We took 5 fields of each group and calculated the distance by Image-pro plus 4.5 software program.

**Immunohistochemistry.**

Immunohistochemistry assay was used for SD rat carotid artery balloon injury model *in vivo*. All arteries were harvested at 3, 14 day after insertion of sildenafil with osmotic pump at 0.8㎎/day. Primary antibodies were rabbit anti-phosphodiesterase 5 (1:100 dilution, Santa Cruz Biotechnology, sc-32884), rabbit anti-cGKΙ α/β (1:100 dilution, Santa Cruz Biotechnology, SC-271765), mouse anti-calponin (1:100 dilution, Sigma, C2687 ) and bound at 4℃ for overnight. Each stained section was detected by DAB (3, 3’-diaminobenzidine) solution and images were obtained using Olympus microscope.

Various tissue samples were obtained from normal SD rat to identify expression pattern of PDE5 and cGK. Caortid artery, penis, heart, brain, liver, spleen, fat, skeletal muscle were fixed with 4% PFA and embedded with paraffin.

Human heart samples were obtained with informed assent after the approval by the Institutional Review Board (IRB) of Seoul National University Hospital. Human heart tissue staining was performed with rabbit anti-phosphodiesterase 5 (1:100 dilution, Santa Cruz Biotechnology, sc-32884) and rabbit anti-cGK α/β (1:100 dilution, Santa Cruz Biotechnology, sc-271765). Each primary antibody was bound at 4℃ for overnight, and HRP tagged secondary antibody for 2hr at RT. PDE5 and cGK were detected by DAB (3,3'-diaminobenzidine) solution and images were obtained using Leica Microscope.

Elastic stain kit (HT25A-1KT, Sigma Aldrich) was used for Van Gieson staining according to manufacturer's instruction. And intima/media ratio was calculated using Image Pro plus 4.5 version software program.

**Rat carotid artery balloon injury**

Carotid artery was injured with ballooning catheter (Edward Lifescience). External carotid artery was denuded and branch of artery was clamped. Up and down side of vessel were fastened after ballooning 15 times. Sildenafil 0.8㎎/day was inserted to jugular vein for 3 days and 14 days using osmotic pump. Harvested vessels were exposed by axial section and viewed with the fluorescent microscope (Olympus).

**Generation of lent virus expressing mutant cGK Ι.**

cGK active and inactive forms of lent virus were kindly provided from Dr. Deguchi A (Columbia University). cGK Ια S56D changed serine at 56 residue to aspartate, cGK Ιβ S80D changed serine at 80 residue. cGK Ια K390R changed lysine at 390 residue to arginine, cGK Ιβ K405R changed lysine at 40 residue. These mutant forms of cGK cloned to pLL3.7 cloning vector. Lent virus packaging vector, pLP1 (encodes viral core protein and viral replication enzyme), pLP2 (encodes Rev protein), pLP/VSV-G (encodes envelop G glycoprotein) were used for making lent virus. JM109 competent cells (RBC bioscience) were used for expressing each plasmid.

**Platelet aggregation.**

For Ex vivo human platelet aggregation assay, Platelet Rich Plasma (PRP) was obtained from human blood sample. Using sodium citrate tube, blood coagulation was inhibited. Blood samples were centrifuged at 800rpm, 4℃ for 15 minutes. And then, opaque soup was harvested and seeded at 12 well multi-plate. Cells were incubated with EBM media (Lonza, cc-3156) with 10% FBS condition. For analysis of platelet aggregation, Chrono-log aggregometer was used, and ADP 2.5μM was effectively induce platelet aggregation.

Cells were stabilized for 10 minutes at incubator, and then sildenafil was treated for 5 minutes, thrombin for 5 minutes. For protein analysis, Sildenafil 1μM, 10μM and platelet aggregation inducer, thrombin 0.1 Unit/㎖ were used. Each antibody, specific for phosphor-VASP239 (1:2500 dilution, Cell Signaling, #3112S), total VASP (1:2500, Cell Signaling, #3112S), Actin (1:2500, Santa Cruz Biotechnology, sc-1616) was used. Before protein was harvested, cells were observed using fluorescent microscope (Olympus).

For in vivo rat platelet aggregation assay, Sildenafil 0.8mg/day was injected to SD rat for 2 weeks (3 times/week). PRP was obtained from abdominal artery, and ADP 2.5μM was used for platelet aggregation inducer.

**Supplementary Figure S1**


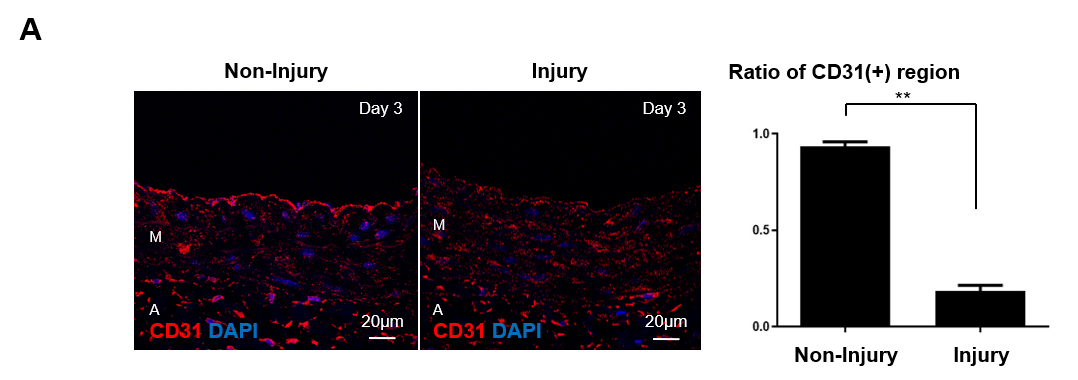


(A) Immunofluorescence staining for CD31 to measure the extent of denudation by balloon injury. Compared to the non-injured group, the balloon-injured group showed much more area for denudation, indication that balloon injury was performed strongly and effectively (n=5). ***P*<0.01.

**Supplementary Figure S2**


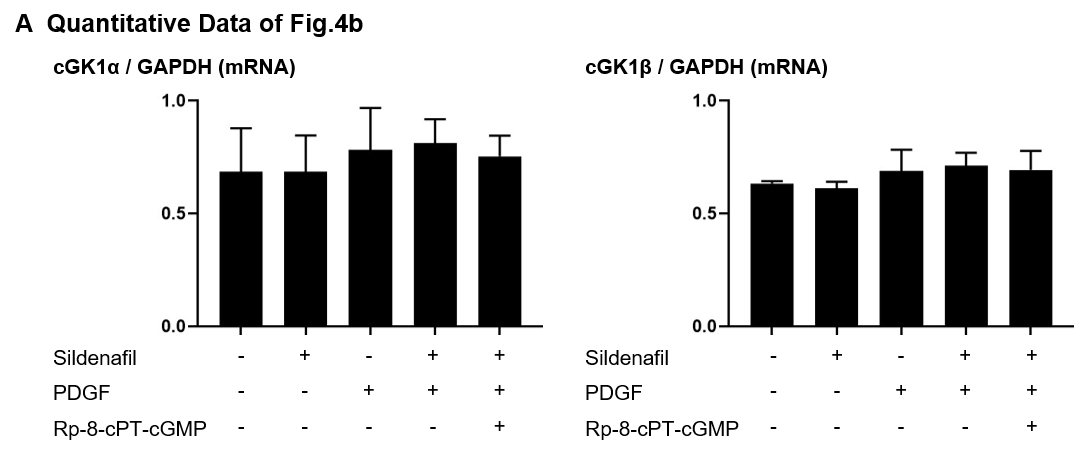


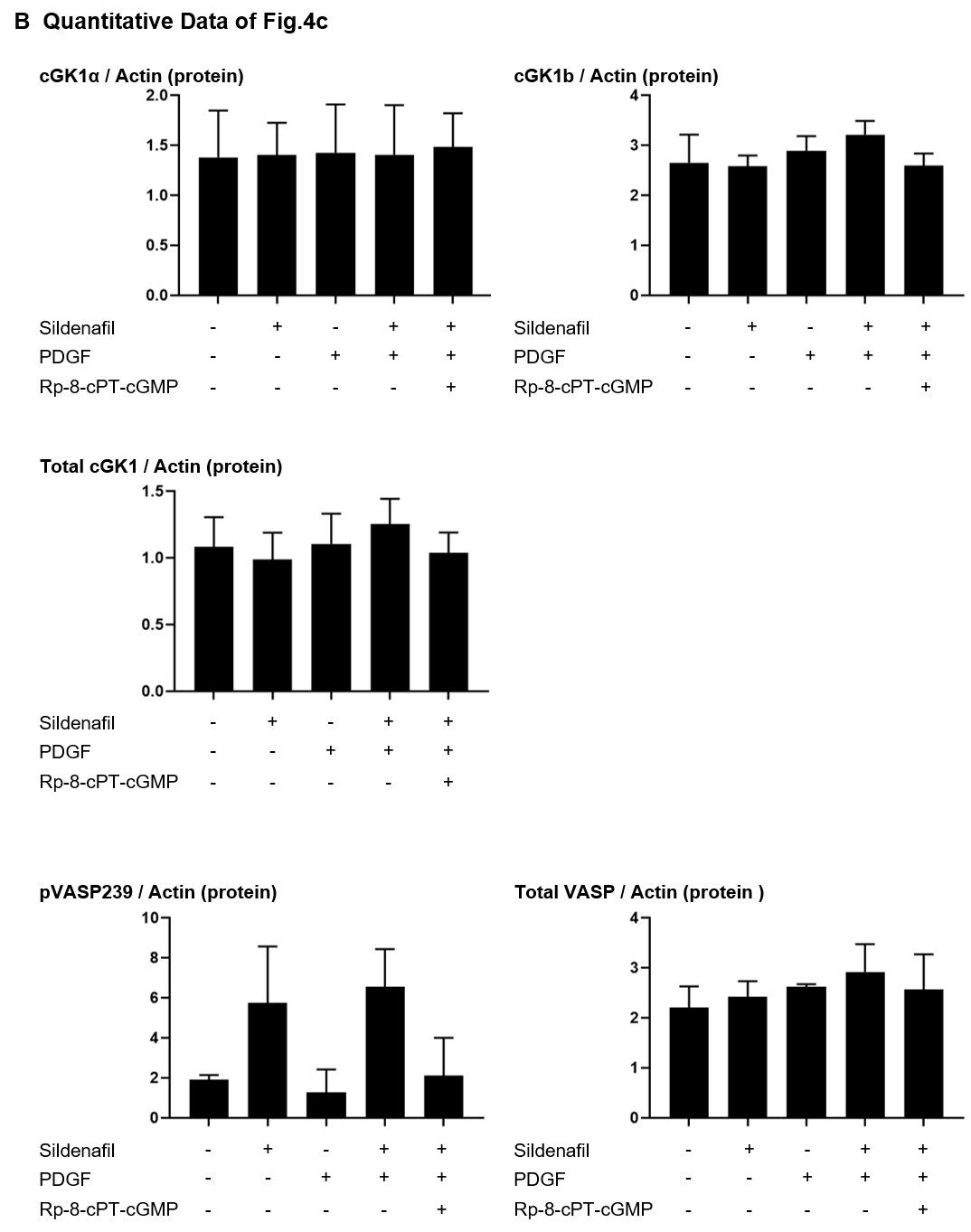


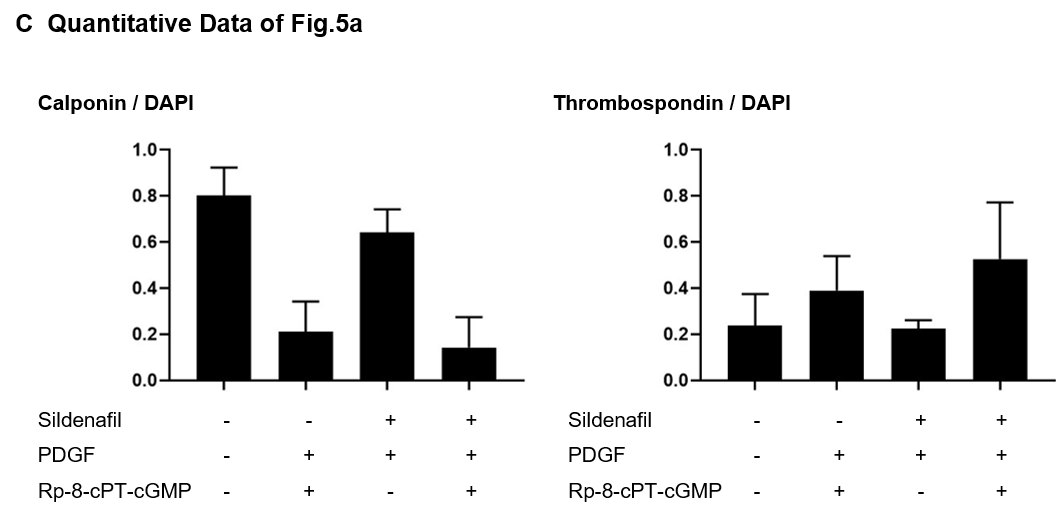


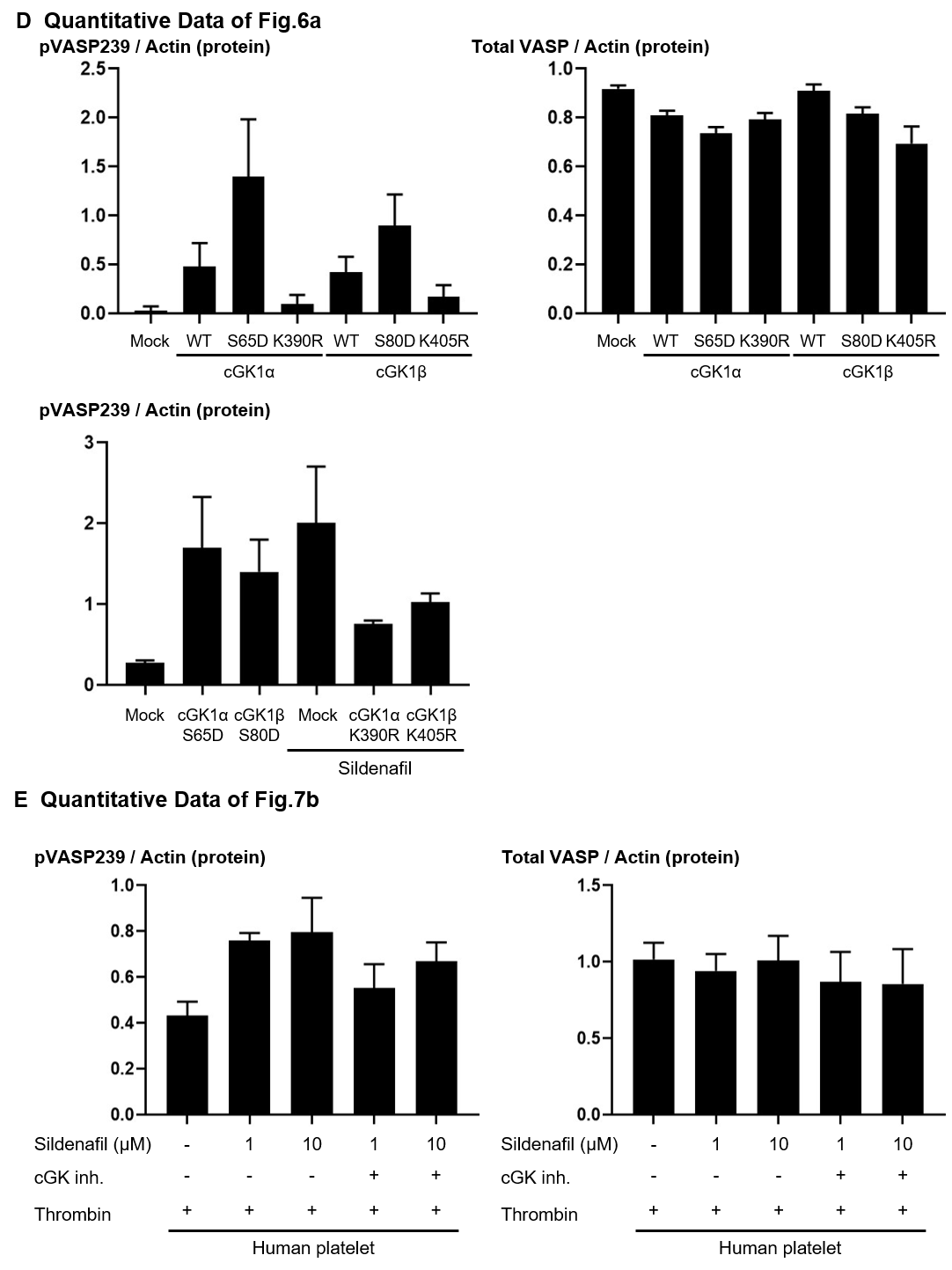

Supplement: Supplementary file 1 — Supplementary Material Online [file 41598_2019_44190_MOESM1_ESM.docx]
